# User Perceptions and Experiences of a Handheld 12-Lead Electrocardiographic Device in a Clinical Setting: Usability Evaluation

**DOI:** 10.2196/21186

**Published:** 2021-08-26

**Authors:** Kam Cheong Wong, Aravinda Thiagalingam, Saurabh Kumar, Simone Marschner, Ritu Kunwar, Jannine Bailey, Cindy Kok, Tim Usherwood, Clara K Chow

**Affiliations:** 1 Westmead Applied Research Centre Faculty of Medicine and Health The University of Sydney Westmead Australia; 2 Westmead Clinical School Faculty of Medicine and Health The University of Sydney Westmead Australia; 3 Bathurst Rural Clinical School School of Medicine Western Sydney University Bathurst Australia; 4 School of Rural Health Faculty of Medicine and Health The University of Sydney Orange Australia; 5 Department of Cardiology Westmead Hospital Westmead Australia; 6 Faculty of Medicine and Health The University of Sydney Sydney Australia; 7 The George Institute for Global Health Sydney Australia; 8 Charles Perkins Centre The University of Sydney Sydney Australia

**Keywords:** handheld, electrocardiogram, ECG, acceptability, usability, user perception, user experience, atrial fibrillation, long QT, screening

## Abstract

**Background:**

Cardiac arrhythmias are a leading cause of death. The mainstay method for diagnosing arrhythmias (eg, atrial fibrillation) and cardiac conduction disorders (eg, prolonged corrected QT interval [QTc]) is by using 12-lead electrocardiography (ECG). Handheld 12-lead ECG devices are emerging in the market. In tandem with emerging technology options, evaluations of device usability should go beyond validation of the device in a controlled laboratory setting and assess user perceptions and experiences, which are crucial for successful implementation in clinical practice.

**Objective:**

This study aimed to evaluate clinician and patient perceptions and experiences, regarding the usability of a handheld 12-lead ECG device compared to a conventional 12-lead ECG machine, and generalizability of this user-centered approach.

**Methods:**

International Organization for Standardization Guidelines on Usability and the Technology Acceptance Model were integrated to form the framework for this study, which was conducted in outpatient clinics and cardiology wards at Westmead Hospital, New South Wales, Australia. Each patient underwent 2 ECGs (1 by each device) in 2 postures (supine and standing) acquired in random sequence. The times taken by clinicians to acquire the first ECG (efficiency) using the devices were analyzed using linear regression. Electrocardiographic parameters (QT interval, QTc interval, heart rate, PR interval, QRS interval) and participant satisfaction surveys were collected. Device reliability was assessed by evaluating the mean difference of QTc measurements within ±15 ms, intraclass correlation coefficient, and level of agreement of the devices in detecting atrial fibrillation and prolonged QTc. Clinicians’ perceptions and feedback were assessed with semistructured interviews based on the Technology Acceptance Model.

**Results:**

A total of 100 patients (age: mean 57.9 years, SD 15.2; sex: male: n=64, female n=36) and 11 clinicians (experience acquiring ECGs daily or weekly 10/11, 91%) participated, and 783 ECGs were acquired. Mean differences in QTc measurements of both handheld and conventional devices were within ±15 ms with high intraclass correlation coefficients (range 0.90-0.96), and the devices had a good level of agreement in diagnosing atrial fibrillation and prolonged QTc (κ=0.68-0.93). Regardless of device, QTc measurements when patients were standing were longer duration than QTc measurements when patients were supine. Clinicians’ ECG acquisition times improved with usage (*P*<.001). Clinicians reported that device characteristics (small size, light weight, portability, and wireless ECG transmission) were highly desired features. Most clinicians agreed that the handheld device could be used for clinician-led mass screening with enhancement in efficiency by increasing user training. Regardless of device, patients reported that they felt comfortable when they were connected to the ECG devices.

**Conclusions:**

Reliability and usability of the handheld 12-lead ECG device were comparable to those of a conventional ECG machine. The user-centered evaluation approach helped us identify remediable action to improve the efficiency in using the device and identified highly desirable device features that could potentially help mass screening and remote assessment of patients. The approach could be applied to evaluate and better understand the acceptability and usability of new medical devices.

## Introduction

Cardiac arrhythmias are a leading cause of death in Australia [1] and internationally [2]. The mainstay method for diagnosing arrhythmias is 12-lead electrocardiography (ECG), and it is commonly used in the community and in primary care to screen and assess for atrial fibrillation and cardiac conduction abnormalities such as prolonged corrected QT interval (QTc) [3]. Atrial fibrillation has been increasing in prevalence and is an important contributor to risk of stroke [4]. A recent systematic review and meta-analysis [5] reported that prolonged QTc is associated with an increased risk of atrial fibrillation. Patients with atrial fibrillation who take antiarrhythmic medication to control their heart rhythm face the potential risk of QTc prolongation, particularly at the start of antiarrhythmic drug therapy [6]. Prolonged QTc is also a marker for long QT syndrome, which increases the risk of sudden cardiac death [7]. Early detection of prolonged QTc in patients would allow clinicians to modify or treat the underlying cause and could potentially reduce the risk of sudden cardiac death [8].

Conventional 12-lead ECG machines have practical limitations to use in community and remote geographic settings due to their bulky size and portability [9]. Portable mobile handheld technologies have a positive impact on accessibility of health care devices at point of care and demonstrate the greatest benefits in contexts where time efficiency and timely clinical decision making are crucial [10]. In the context of timely diagnosis of cardiac abnormalities, there is a need for more portable ECG devices. Handheld or wearable ECG devices (such as AliveCor Kardia, MyDiagnostick, Omron, and the Apple Watch) have become increasingly prevalent in the market. However single-lead handheld ECGs are limited in their ability to detect arrhythmias, and there is minimal evidence of their utilization in clinical practice [3]. Furthermore, most single-lead handheld ECG devices cannot automatically report QTc measurements [11]. QTc measurements in single-lead rhythm strips produced by Apple watches were validated against those from conventional 12-lead ECGs to enable remote assessment of patients [12]. Remote assessment was particularly important during the coronavirus disease 2019 pandemic. Clinicians manually measured and calculated the QTc using the single-lead ECG trace [12]. Manual calculation of QTc is time-consuming, particularly when QTc varies with variation in heart rate caused by change in body position (supine and standing) [13,14]. Portable 12-lead ECG devices that automatically report QTc and other ECG parameters could improve clinicians’ ability to diagnose prolonged QTc and other cardiac abnormalities.

In tandem with an increasing number of technology options to acquire ECG in various clinical settings, evaluation of device usability should go beyond validation of the device in a controlled laboratory setting. A recent review [15] on mobile health technology acceptance reported that assessment of users’ experiences is crucial because assessments of user experiences provide insights and opportunities to improve the device, and user experiences can affect intention to use the device. However, common approaches for evaluating medical devices [16] were assessing product performance at research and development stage and compliance with regulatory requirements and they lack focus on user training, lack focus on efficiency in using the device, and lack assessment of users’ perceptions and experiences in clinical settings.

We aimed to pragmatically evaluate ECG devices within the setting in which they will be used, by assessing (1) device reliability in producing key ECG parameters and diagnosing atrial fibrillation and prolonged QTc for 2 patient postures (supine and standing) in a clinical setting, (2) user time efficiency, (3) patients’ experiences with the device, and (4) clinicians’ perceptions of and feedback on potential use of the device for clinician-led mass screening.

## Methods

### Study Design, Setting, and Participants

**Figure 1:**
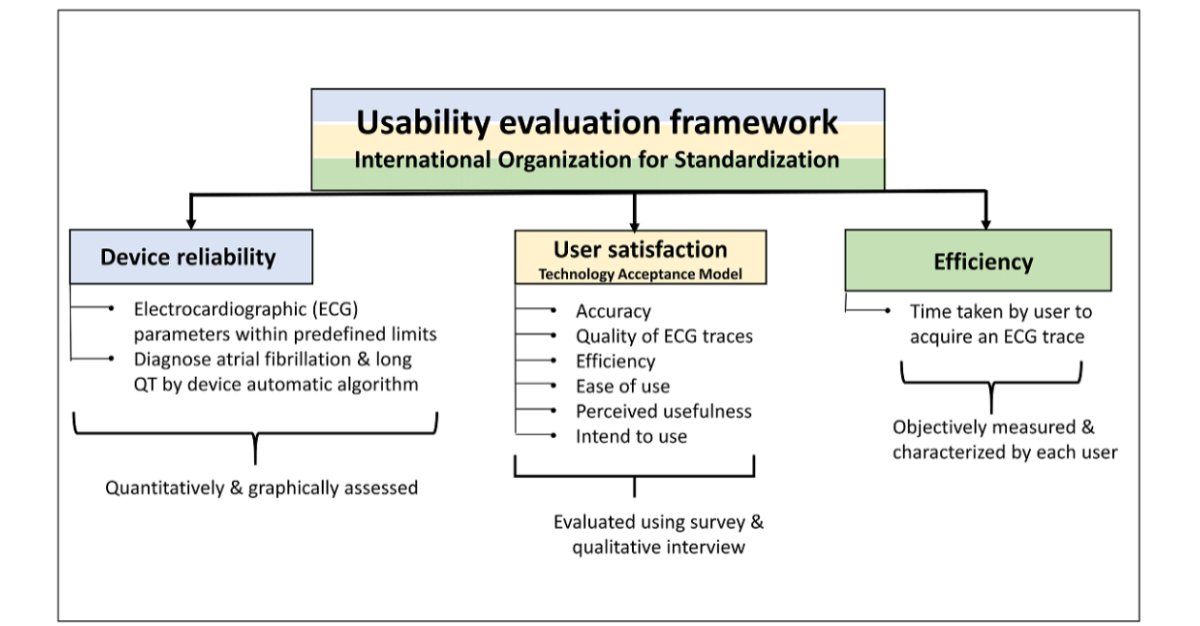
Usability evaluation framework.

We used a mixed methods approach [17] to evaluate device usability. The framework (Figure 1) for assessing device usability was based on International Organization for Standardization Guidelines on Usability [18] and included reliability of the device, efficiency when using the device, and user satisfaction with the device [19].

The study was conducted in an outpatient cardiology clinic and inpatient cardiology ward at Westmead Hospital, New South Wales, Australia. The hospital setting was selected because patients with various arrhythmias and cardiac diseases present to the hospital and require ECG assessment. Patients (age ≥18 years) and their clinicians were recruited. Patients who were too ill or unable to provide consent were excluded. Users were clinicians who applied the device to acquire ECGs and patients who were connected to the device.

### Handheld and Conventional 12-Lead ECG Devices

**Figure 2:**
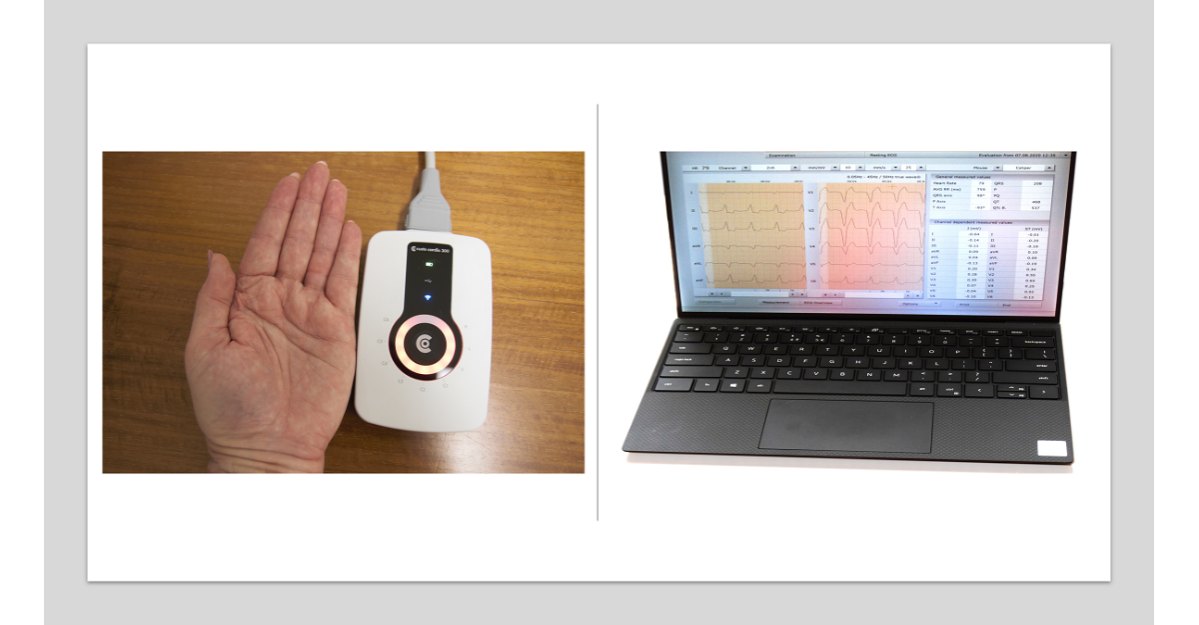
Handheld electrocardiography device and graphical interface on a laptop computer.

A handheld 12-lead ECG device (Cardio 300, custo med GmbH [20]; Australian Register of Therapeutic Goods number 302423) (Figure 2) was selected because of its small size (length by width by thickness: 11.5 cm × 7.5 cm × 1.8 cm; weight: 430 g) and ability to transmit ECG data via Bluetooth. It was compared with the routinely used conventional 12-lead ECG machine at each clinic site—Mortara Eli 280 (Welch Allyn Inc) in the outpatient cardiology clinic and Mac 5500 (General Electric) in the inpatient cardiology ward.

### Reliability

Device reliability [21] is defined as its ability to reproduce measurements (QT interval, QTc interval, heart rate, PR interval, and QRS interval) consistently within an acceptable limit in a clinical setting. We chose QTc interval as the primary measurement because of its importance in assessing prolonged QTc—which is defined as an individual QTc measurement ≥460 ms in females and an individual QTc measurement ≥450 ms in males [5]. There are guidelines for an acceptable limit of variation in QTc measurements [22]. A clinically noteworthy change in QTc from baseline is defined as 30 ms to 60 ms [22,23]. We defined acceptable limits of mean difference for QTc as within ±15 ms. Both devices produced QTc readings using the Bazett formula [24]. Agreement between devices’ automatic ECG interpretation algorithms for diagnosing atrial fibrillation was also assessed.

### Efficiency

Efficiency was measured as the total time taken by the clinician to place electrodes on a patient and acquire the first ECG trace. A research assistant measured the time taken by the clinician to place electrodes while the patient was supine. The electrodes were left in place on the patient during device change over. The time taken by the clinician to connect each ECG device to the electrodes and acquire the first ECG trace was measured separately for each device.

### User Satisfaction

Clinician satisfaction was measured using 5-point Likert scales (for device accuracy, quality of ECG traces, ease of use, and efficiency). In addition, semistructured interviews were conducted for feedback and to assess clinicians’ acceptance of the device; semistructured interview guides were based on the Technology Acceptance Model [25,26] (ie, ease of use, perceived usefulness, and intention to use the device). Patient satisfaction with the device connected to them was assessed using a 5-point Likert scale.

### Sample Size

The required sample size of patients was calculated (Sealed Envelope, Sealed Envelope Ltd) based on the primary objective to evaluate device reliability using within- and between-device variabilities in producing QTc measurements within the predefined acceptable limit of ±15 ms. With a power of 80% and α=5%, the required sample size was 96, which we rounded up to 100.

### Data Collection Procedures

A research assistant provided in-person training to clinicians to demonstrate how to use the handheld device to acquire ECGs. In addition, clinicians could opt to watch a short introductory video about how to use the handheld device. For each patient, ECGs were acquired twice while supine and while standing using each device. ECG acquisition order was randomized with block sizes of 2 and 4 using SAS (version 9.4; SAS Institute). The randomized sequences, participants’ demographic data and ECG parameters (QT, QTc, heart rate, PR and QRS) were recorded in REDCap [27].”

### Survey Questionnaire and Semistructured Interview

Patient’s satisfaction while connected to the devices were surveyed using 5-point Likert scale (1, strongly disagree to 5, strongly agree). Before using the handheld device, clinicians were asked whether they had previously used a similar device (yes or no); if yes, we asked the name of the device. Using 5-point Likert scales, the clinicians rated importance (1, strongly disagree to 5, strongly agree) and satisfaction (1, not satisfied to 5, very satisfied) with respect to accuracy, quality of the ECG trace, ease of use, and efficiency. We also asked clinicians whether they found the handheld device easier to use than the routinely used conventional ECG machine, whether using the handheld device in their current workflow could increase their productivity, and assuming they had continual access to the handheld device, whether they intended to use it. In semistructured interviews, we asked clinicians if they found the handheld device useful and to explain their response, if their needs were met when using the handheld device (probing questions: what were their needs and what could the device do to better serve their needs), and if the handheld device could be applied for clinician-led mass screening (probing question: how to make the device suitable for clinician-led mass screening?).

### Statistical Analysis

Demographic data are presented using descriptive statistics. Reliability, in terms of agreement between devices in diagnosing atrial fibrillation and prolonged QTc, was assessed using the κ statistic [21], for which κ=0.41 to κ=0.60 is generally considered to demonstrate moderate agreement and κ>0.61 considered to demonstrate good agreement. Within- and between-device reliability in QTc measurements was assessed using the intraclass correlation coefficient (ICC); ICC ≥0.7 demonstrates good reliability [21]. The within-device variability for QTc was assessed by the difference in QTc in the first and second ECG acquired immediately one after another by the same device on the same patient. The between-device variability was assessed by the difference in QTc in the first ECG produced by the 2 devices. The between-device variability over a range of QTc intervals was examined using Bland-Altman plot [28]. The within-device and between-device variabilities in QTc compared with the predefined acceptable limits of ±15 ms were examined by plotting the mean of the differences in QTc and their 95% confidence intervals in forest plots. Similarly, within-device and between-device variability in other key ECG parameters (QT interval, heart rate, PR interval, and QRS interval) were examined using forest plots.

The differences in clinicians’ ECG acquisition times using the devices were assessed using a scatter plot. A logarithmic transformation was used for the frequency of usage because the time difference due to a unit of increment of usage from first to second usage was not proportional to a unit of increment in subsequent usages (ie, the change in time difference levelled off as the frequency of usage increased). These time differences were analyzed using linear regression analysis. The impact of the randomized sequence of using the devices on the ECG acquisition times was analyzed using a 2-tailed *t* test. Quantitative data were analyzed using SPSS statistical software (version 25; IBM Corp), except linear regression analysis, which was performed using R software (R Foundation for Statistical Computing). Normality of distribution was assessed with a Shapiro-Wilk test [29]. Nonparametric testing (Wilcoxon signed ranked test using the Hodges-Lehman method to compute 95% CI of the median difference) was used for nonnormal distributions. A *P* value <.05 was considered significant.

Semistructured interviews were recorded and transcribed verbatim for inductive thematic analysis. Two investigators (KW and JB) coded the interview transcripts independently, generated a draft codebook, and then convened to reach consensus on the final codebook. Any discrepancy in coding was resolved by discussion. Interview transcripts were thematically analyzed using NVivo (version 12; QSR International).

### Ethics

The study was approved by Western Sydney Local Health District Human Research Ethics Committee (ethics approval number 5929).

## Results

### General

A total of 100 patients were recruited and participated from July to December 2019. The mean age of patients was 57.9 years (SD 15.2). Participant demographics, morbidities, and medication profiles are shown in Table 1.

**Table 1:** Characteristics of the patients.

Characteristic				Total (n=100)
**Sex, n (%)**				
	Male			64 (64)
	Female			36 (36)
**Age (years)**				
	Mean (SD)			57.9 (15.2)
	Range			18-88
	Median (IQR)			61.0 (20.0)
**Preexisting morbidity^a^** **,** **n (%)**				
	**Heart diseases**			
		Ischemic heart disease	27 (27)	
		Cardiomyopathy	7 (7)	
		Valvular disease	6 (6)	
		Heart blocks	6 (6)	
		Pacemaker	6 (6)	
	**Arrhythmia**			
		Atrial fibrillation	14 (14)	
		Paroxysmal atrial fibrillation	3 (3)	
		Atrial flutter	3 (3)	
		Supraventricular tachycardia	1 (1)	
	Hypertension			63 (63)
	Hypercholesterolemia			42 (42)
	Diabetes			27 (27)
**Medications^a^** **,** **n (%)**				
	Antihypertensive medication			53 (53)
	Anticoagulant/antiplatelet			47 (47)
	Lipid lowering medication			43 (43)
	Oral hypoglycemic			18 (18)
	Diuretics			15 (15)
	Antiarrhythmic medication			9 (9)
	Insulin			3 (3)

^a^The total exceeds 100% because many patients had more than 1 morbidity or took more than 1 medication.

A total of 11 clinicians (nursing staff: n=10; clinical trial coordinator: n=1) participated. Prior ECG experience was high, with 10 of the clinicians routinely acquiring ECGs daily or weekly and 1 clinician routinely acquiring ECGs fortnightly. Among the clinicians, 8 were from outpatient cardiology clinics, and 3 were from inpatient cardiology wards (Table 2). Most clinicians (9/11, 82%) opted to receive a demonstration of how to use the handheld device from the research assistant while the remaining 2 clinicians (clinicians 1 and 6) opted to watch a short video demonstration. Clinicians 1, 2, 3, and 6 were nurses working in cardiology outpatient clinics, and they acquired ECGs daily and recruited 18, 16, 19, and 20 patient participants, respectively.

**Table 2:** Characteristics of the clinicians.

Characteristic				Total (n=11)
**All, n**				
	Outpatient		8	
	Inpatient		3	
**Role,** **n (%)**				
	Nurse		7 (63.6)	
	Nurse educator		3 (27.3)	
	Clinical trial coordinator		1 (9.1)	
**Gender,** **n (%)**				
	Male		4 (36)	
	Female		7 (64)	
**How often do you acquire an ECG^a^? n (%)**				
	Daily		6 (55)	
	Weekly		4 (36)	
	Fortnightly		1 (9)	
	Monthly		0 (0)	
**Have you used the 12-lead handheld ECG device before? n (%)**				
	Yes		0 (0)	
	**No. If no, have you used similar device before? n (%)**		11 (100)	
		Yes^b^	1 (9)	
		No	10 (91)	
**Number of patients recruited per clinician**				
	Mean		9	
	Median		6	
	Range		1-20	

^a^ECG: electrocardiography.

^b^Clinician 10 had used wireless electrocardiography before but not the handheld device used in this study.

**Figure 3:**
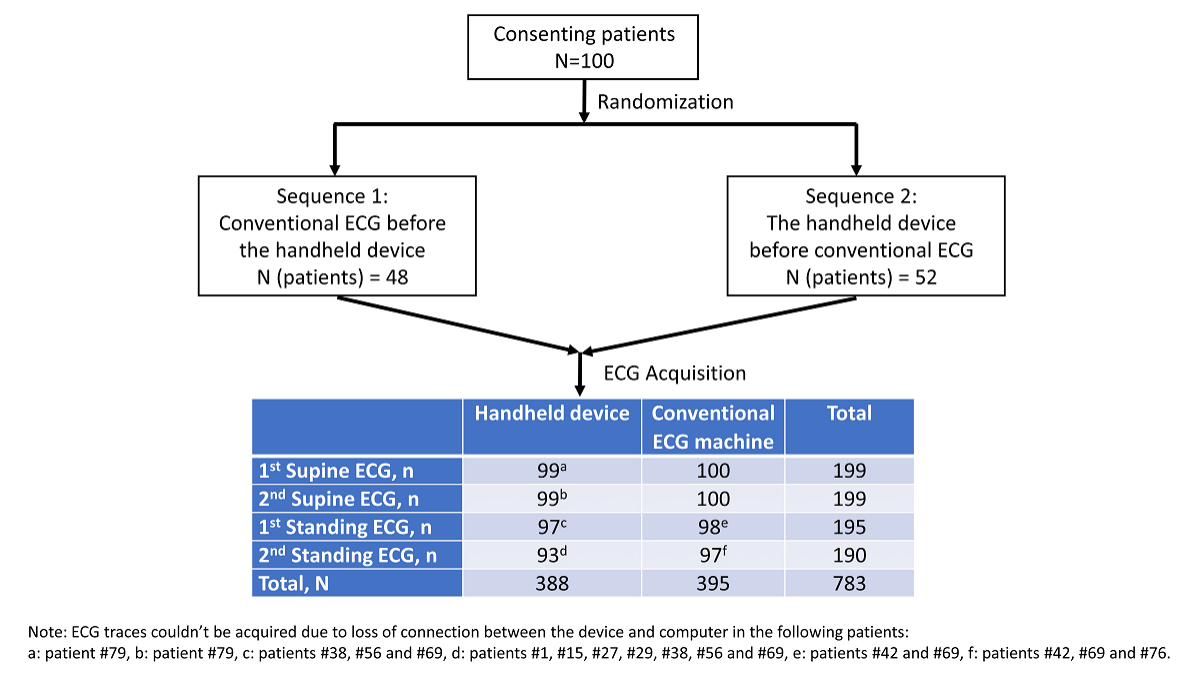
Patient randomization and number of electrocardiograms (ECG) acquired. ECG traces could not be acquired due to loss of connection for (a) 1 patient, (b) 1 patient, (c) 3 patients, (d) 7 patients, (e) 2 patients, and (f) 3 patients.

A total of 783 ECGs were collected (Figure 3). The randomized sequence for ECG device order resulted in the handheld device being connected first for 52 patients and the conventional ECG machine being connected first for 48 patients.

### Reliability

**Figure 4:**
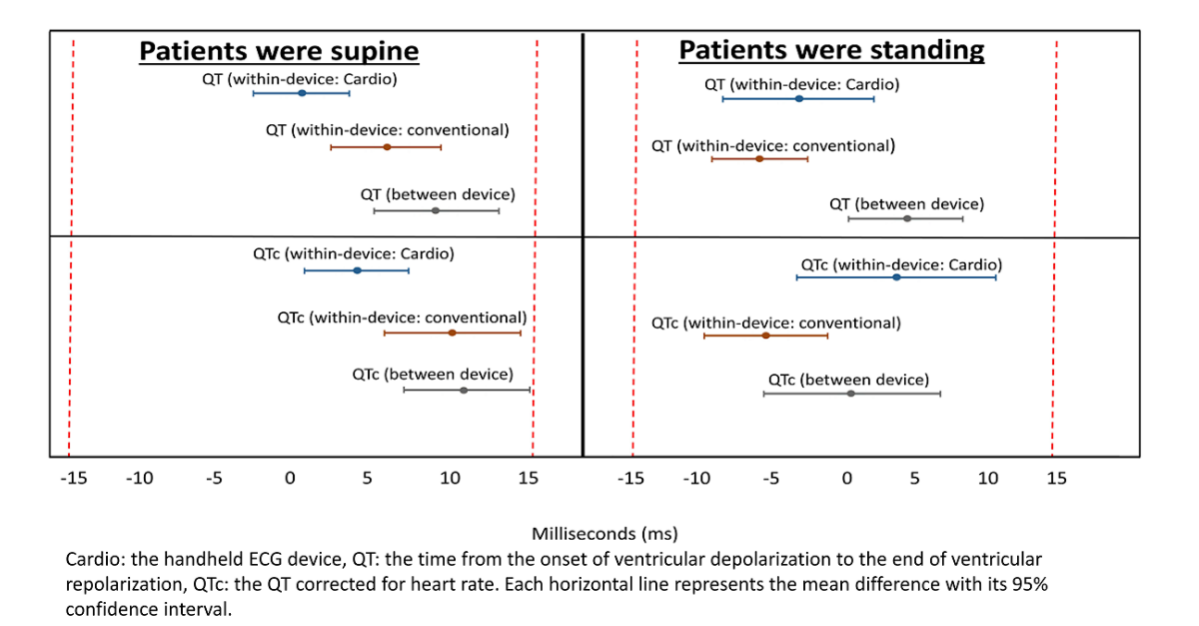
Within- and between-device variability of QT interval and corrected QT interval by patient posture. Mean differences and 95% confidence intervals. Dashed red lines indicate predefined acceptable limits.

The within- and between-device mean differences for QT and QTc while patients were supine and standing were consistently within ±15ms for both handheld and conventional devices (Figure 4).

**Figure 5:**
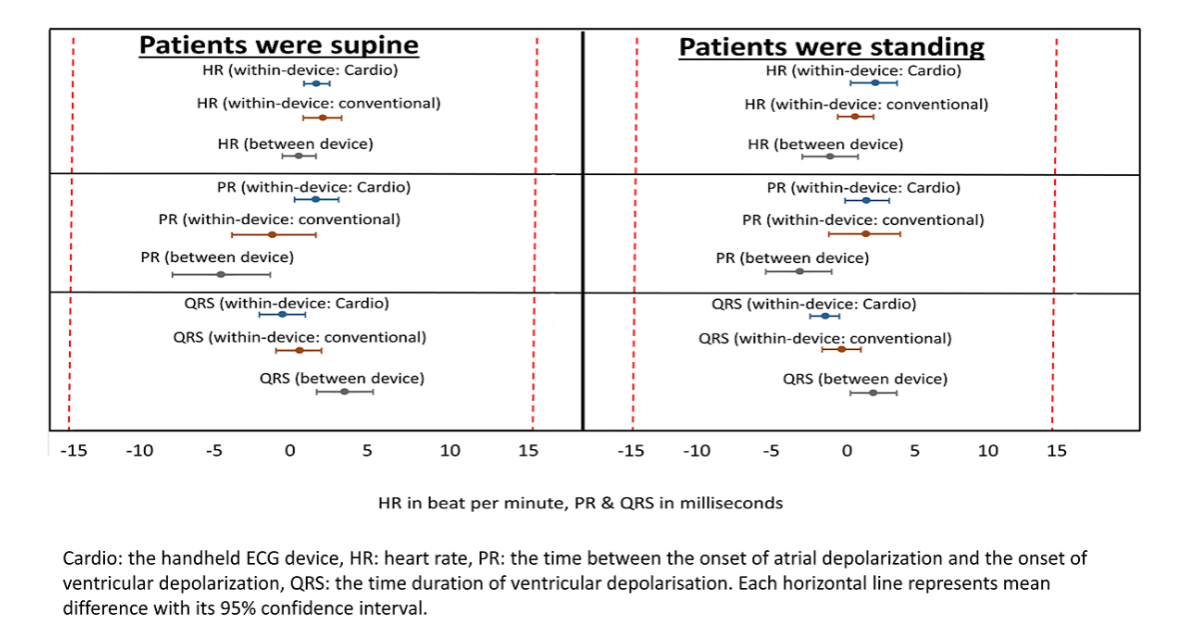
Within- and between-device variability of heart rate, PR interval, and QRS interval by patient posture. Mean differences and 95% confidence intervals. Dashed red lines indicate predefined acceptable limits.

Both devices had good reliability in producing heart rate, PR interval, and QRS interval measurements while patients were supine and standing. The within- and between-device mean differences in heart rate, PR interval, and QRS interval measurements were ±5 bpm, ±10 ms, and ±10 ms (Figure 5).

**Figure 6:**
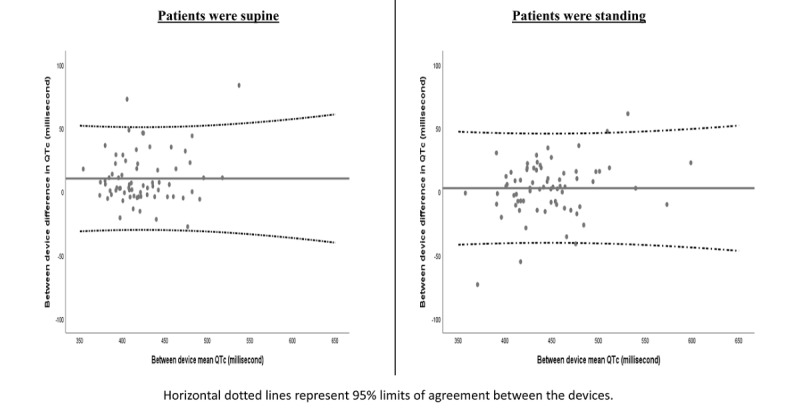
Bland-Altman plots for differences between (first handheld device and conventional) individual corrected QT interval measurements.

Variability of the differences in QTc measurements between the handheld device and conventional ECG machines were randomly distributed (Figure 6). For conventional ECG, the difference between standing (median 436.4 ms, IQR 46.0 ms) and supine QTc measurements (median 410.3 ms, IQR 49.0 ms) was significant (*P*<.001). The median of the difference between QTc standing and QTc supine was 20.7 ms (95% CI 15.3-25.6). For the handheld device, the difference between standing (median 446.0 ms, IQR 50.0 ms) and supine QTc measurements (median 420.0 ms, IQR 48.0 ms) was significant (*P*<.001). The median of the difference between QTc standing and QTc supine was 14.5 ms (95% CI 10.5-19.0).

The devices had good agreement in diagnosing atrial fibrillation and prolonged QTc (within- and between-device κ=0.68-0.93) (Table 3). The within- and between-device reliabilities for QTc measurements were high (ICC 0.90-0.96) (Table 3).

**Table 3:** Within- and between-device agreement in diagnosing atrial fibrillation and prolonged QTc. Reliability of QTc measurements.

Variable^a^			Within-device κ or ICC^b,c^ (95% CI)					Between-device κ or ICC^c^ (95% CI)
		Handheld device		Conventional machine				
**Atrial fibrillation**								
	Patients were supine	0.82 (0.58-1.00)		0.79 (0.50-1.00)		0.90 (0.71-1.00)		
	Patients were standing	0.68 (0.40-0.97)		0.84 (0.62-1.00)		0.93 (0.78-1.00)		
**Prolonged QTc^d^**								
	Patients were supine	0.84 (0.68-0.99)		0.75 (0.55-0.94)		0.92 (0.81-1.00)		
	Patients were standing	0.71 (0.54-0.88)		0.77 (0.61-0.93)		0.69 (0.51-0.86)		
**QTc measurements**								
	Patients were supine	0.96 (0.93-0.97)^c^		0.92 (0.88-0.95)^c^		0.92 (0.88-0.95)^c^		
	Patients were standing	0.90 (0.84-0.94)^c^		0.95 (0.92-0.97)^c^		0.94 (0.90-0.96)^c^		

^a^Clinicians mistakenly reprinted the second conventional ECG from the first ECG in the first 20 patients, and 8 other patients declined repeating ECG acquisition after several attempts. These 28 patients were excluded from this analysis resulting in a sample size of 72.

^b^ICC: intraclass correlation coefficient.

^c^These values are ICCs.

^d^QTc: corrected QT interval.

### Time Efficiency

**Figure 7:**
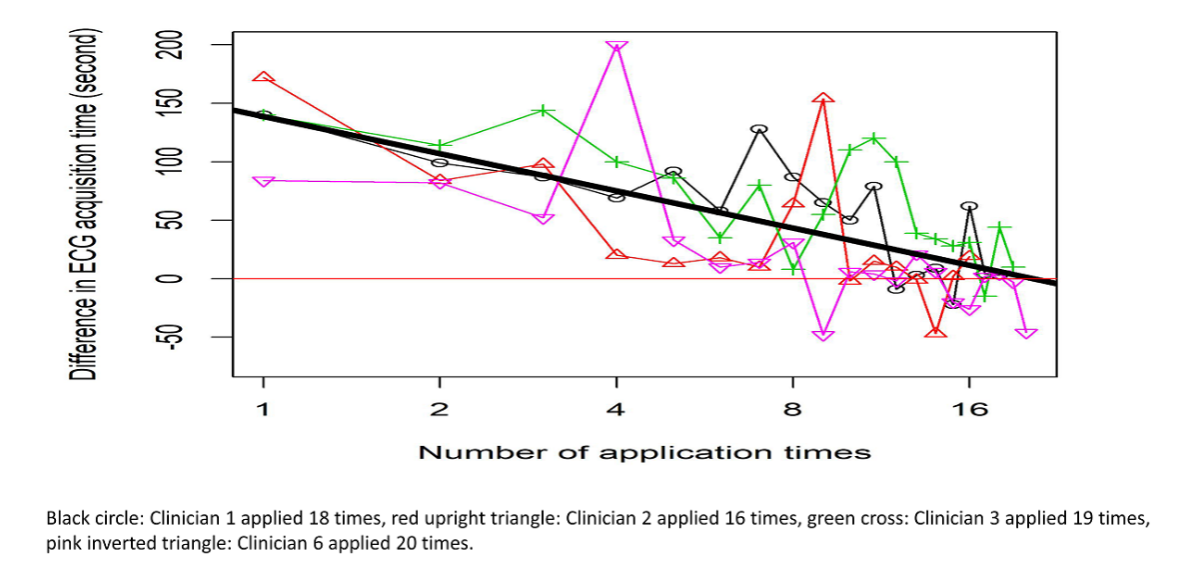
Difference in the time taken by clinicians to acquire 12-lead electrocardiography measurements using the handheld device and conventional machine by usage frequency.

The mean time taken to place electrodes on patients (regardless of ECG device) was 42.2 seconds. The mean times taken by clinicians to acquire the first ECG using the handheld device and conventional ECG machine were 144.6 seconds and 103.5 seconds, respectively. On average, the total times taken by clinicians to acquire an ECG using the handheld device and conventional ECG machine were 186.8 seconds and 145.7 seconds. The median of the difference between the clinicians’ ECG acquisition time using the handheld device and conventional ECG machine was 39.5 seconds (95% CI 27.0-51.0). These times excluded the time taken to prepare the patient (eg, the time taken by the patients to undress themselves for the procedures). The randomized sequence of applying the devices had insignificant effect on the difference in ECG acquisition time (conventional: *P*=.51; handheld: *P*=.97). ECG acquisition times improved with the number of time clinicians used the devices (*P*<.001) (Figure 7). The difference in clinicians’ ECG acquisition times using the devices approached 0 after clinicians had used the device 18 times (Figure 7).

### User Satisfaction and Acceptance

**Figure 8:**
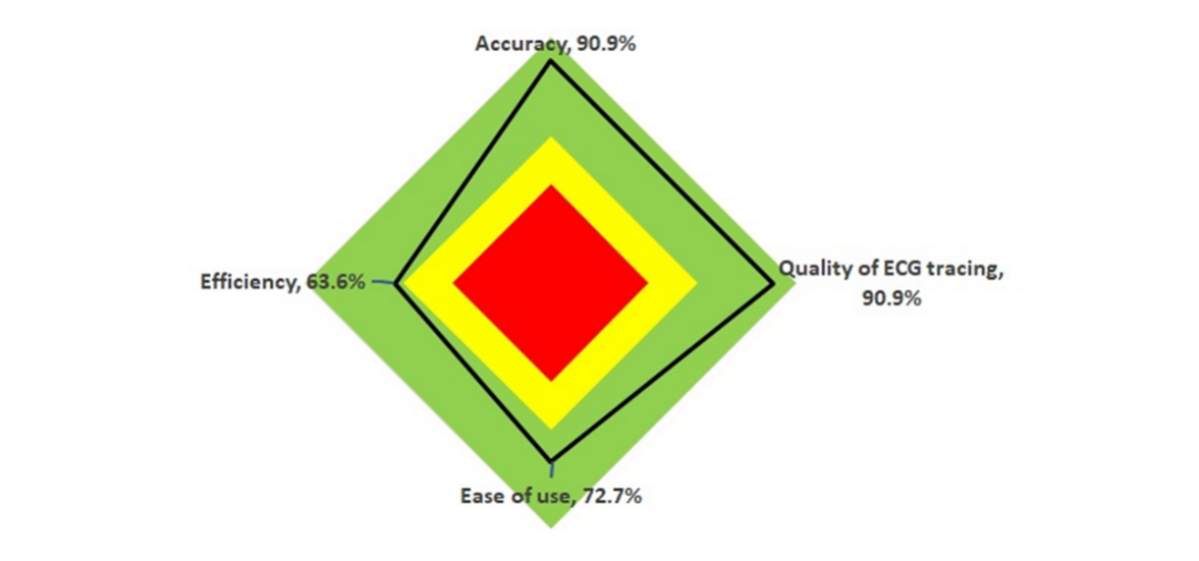
Radar chart of clinician satisfaction after using the handheld device. ECG: electrocardiogram.

Clinicians’ expectations of the handheld device before and satisfaction after are shown in Table 4 and Figure 8. Patients’ experiences are also shown in Table 4.

**Table 4:** Clinician and patient satisfaction.

Survey item					Rating, mean (SD)
**Clinicians’ expectations before using the handheld device and their satisfaction after using it**					
	**Accuracy**				
		Before^a^	5.0 (0.0)		
		After^b^	4.0 (0.8)		
	**Quality of ECG^c^** **trace**				
		Before^a^	5.0 (0.0)		
		After^b^	4.0 (0.8)		
	**Ease of use**				
		Before^a^	4.7 (0.5)		
		After^b^	3.9 (0.9)		
	**Efficiency**				
		Before^a^	4.9 (0.3)		
		After^b^	3.5 (1.0)		
**Clinicians’ response after using the handheld device**					
	Compared to conventional ECG, I found the handheld device easier to use^d^			3.4 (0.8)	
	Using the handheld device in my job increased my productivity^d^			3.1 (0.5)	
	Assuming I had continual access to the handheld device, I intended to use it^d^			3.6 (0.5)	
**Patients’ experience with the handheld device compared to the conventional ECG machine**					
	**Patient felt comfortable while connected to the device and lying down**				
		The handheld device^d^	3.2 (0.6)		
		Conventional ECG^d^	3.0 (0.3)		
	**Patient felt comfortable while connected to the device and standing up**				
		The handheld device^d^	3.2 (0.5)		
		Conventional ECG^d^	3.1 (0.4)		

^a^5-point Likert scale ranging from 1 (not important) to 5 (very important).

^b^5-point Likert scale ranging from 1 (not satisfied) to 5 (very satisfied).

^c^ECG: electrocardiography.

^d^5-point Likert scale ranging from 1 (strongly disagree) to 5 (strongly agree).

### Thematic Analysis of Interviews With Clinicians

#### Overview

Most (10/11) clinicians attended a 1-to-1 interview. One clinician was on leave and could not attend the interview. When asked about their needs, satisfaction, and useful features of the handheld device, clinicians frequently mentioned the following features: quality of the ECG traces, ease of use, efficiency, accuracy, small size, and portability.

#### Main Themes

When asked about their needs regarding an ECG device, clinicians expressed that they wanted a device that was easy to use, efficient (took short time to acquire an ECG), accurate, and produced good-quality ECG traces. Clinicians reported satisfaction with the accuracy and quality of the ECG traces produced by the handheld device, however, were equivocal about the efficiency due to the extra time they took to acquire an ECG.

My needs are to do ECG quick and efficient as possible, and better quality ECG. Quality-wise it is good, but I am slightly satisfied with the efficiency of the device.

Clinician 1

I am satisfied with the quality and accuracy of the device. I also find it easy to use. But usually, it takes more time than the old device.

Clinician 6

My needs are to acquire accurate and better quality ECGs. I am satisfied with accuracy, efficiency and quality of the device.

Clinician 8

Feedback about interference (loss of connection between the device and computer) varied between clinicians in cardiology inpatient wards and outpatient clinics. Clinicians in inpatient wards reported less interference in the handheld device than that in a conventional ECG machine, however, clinicians in outpatient clinics found the opposite.

There is a lot of interference with the connection of the device. The connection gets lost multiple times in between while doing ECGs, especially for standing one.

Clinician 3, cardiology outpatient clinic where patients had ECG acquired before seeing cardiologist

It has less interference mainly while doing standing ECGs compared to conventional device.

Clinician 8, cardiology inpatient ward

The aspects that I liked about this device is it’s clear, less interference with cardiac monitors, small, light-weight, easy to carry and chargeable.

Clinician 11, cardiology inpatient ward

Clinicians specified portability as a desired feature of the handheld ECG device. The wireless transmission of ECG trace from the device to a laptop computer was an added advantage; however, the need to carry a laptop computer to connect with the ECG device could be a drawback.

I do find it useful as far as portability wise it is good. However, it takes more time compared to the old device. Few aspects of the device, I like are it's handy, easy to carry, save lots of space and ECG are saved within the laptop so less chance of loss of ECG. Also, no damage to papers and no extra cost for papers.

Clinician 1

I like the most about this device is it is portable, small and quite fast picking up ECG sometimes. But, most of the times we need to wait a few more seconds to get a satisfactory ECG. The thing I don't like about this device is it is attached to the laptop. The device is smaller and easy to carry everywhere, but along with this, we should also carry a laptop everywhere. I feel for our clinic (Rapid Access Cardiac Clinic) paper is more efficient.

Clinician 6

Most clinicians (9/11) agreed that the handheld device was suitable for clinician-led mass screening; 1 clinician was unsure, and 1 clinician stated “yes” with suggested enhancements to the device. Suggestions for enhancements included improving efficiency in using the device to acquire ECG by increasing user training,

As it is small in size and not bulky and easy to carry everywhere, it can be used for mass screening. But, firstly, clinicians who will be using this device for mass screening purpose should be properly and adequately trained.

Clinician 3

and improving wireless transmission of ECG trace to a computer (increasing the range of wireless transmission to allow patient isolation for infection control and remote assessment of patients).

Long range would help for infection control use. ie receiver on patient and device outside the room for an isolated patient.

Clinician 10

It is a small device so we can easily carry this device everywhere, even in remote areas. So, I think it is more convenient for mass screening in rural settings where it is difficult to carry the old device.

Clinician 2

## Discussion

### Principal Findings

The results suggested that the handheld device had high reliability in producing key ECG parameters and had good levels of agreement with the conventional ECG machines in diagnosing prolonged QTc and atrial fibrillation using the device automatic algorithm. The clinicians’ efficiency in using the devices improved with usage, which was demonstrated by ECG acquisition times. This user-centered approach helped us identify remediable action to improve user efficiency with training. Highly desirable device features, such as portability (small size and lightweight) and wireless ECG transmission (enhancement in the wireless range of ECG transmission from the device to computer) allow clinician-led mass screening and remote assessment of patients to be feasible. However, the mass screening should be clinician-led because users require the skill to apply electrodes correctly on the body.

The mixed methods approach allowed us to explore diverse perspectives in the usability of the handheld device. Quantitative evaluation of clinicians’ ECG acquisition times provided an objective measurement of time efficiency and characterized the trend of improvement in efficiency with the number of usage (Figure 7). In this study, we found that differences in ECG acquisition times between the devices approached 0 after the users used the devices 18 times. Quantitative measurements of ECG acquisition times revealed the learning curves of different users. With qualitative evaluation of user perceptions and experiences in using the device, we identified their training needs, desired device features, and suggestions to make the device suitable for clinician-led mass screening and remote assessment of patients. This mixed methods approach addressed gaps in the common approaches to medical device evaluation, which lack evaluation of user perceptions, experiences, and efficiency [16].

The devices had high reliability in producing key ECG parameters while patients were supine and standing. This was consistent with findings in previous research. Madias and colleagues [30] evaluated a standard 12-lead ECGs recorded in patients in supine and standing positions and concluded that the ECG results in supine and standing were comparable. This comparability may allow ECG recording in busy clinical setting to be more cost-effective—ECGs could be acquired while patients are standing. Despite high reliability, we should note that QTc measurements (regardless of device) while patients were standing compared to those when supine were longer, which was consistent with the literature [13,14]. Compared with QTc measurement when supine, QTc measurement while standing was more accurate in distinguishing patients with long QT syndrome from individuals without long QT syndrome [14]. Lengthened QTc while standing could assist clinicians to diagnose long QT syndrome. Researchers should evaluate the effect of change in body position when comparing device reliability.

Clinician perceptions of efficiency were affected by their familiarity with the device. It was expected that users would take a longer time using the newly introduced handheld device to acquire an ECG than when using a familiar conventional ECG machine because users lacked familiarity with the new device. It is worthwhile to note that the time measured in this study excluded the time to prepare the patients (eg, time for patients to remove their top clothing and get ready for the procedure). The clinicians’ ECG acquisition times were less than the 10.6 minutes reported by Somerville and colleagues [31] (which included the time taken for preparing the patients) using conventional 12-lead ECG in general practice. The clinicians in our study were mainly nursing staff working in the cardiology clinic and ward, and they acquired ECGs daily or weekly. The clinicians’ familiarity with acquiring ECG could differ in comparison to those in general practice setting, and this factor should be taken into consideration when formulating a training program.

Clinician experience in using the device was contextual. In the inpatient cardiology ward, clinicians reported that there were less artefacts on the ECGs because of less interference between the handheld device and the other monitors to which patients were connected, but clinicians in the outpatient cardiology clinic reported that there were several incidences of lost Bluetooth connection between the handheld device and laptop computer resulting in multiple attempts to reacquire ECGs. Thus, the experiences and resulting perceptions on ease of use of the device varied depending on the environment in which the device was used. Patient satisfaction was mainly focused on their comfort when connected to the devices. Most of the patients felt comfortable during ECG acquisition with both handheld and conventional devices.

### Strengths and Limitations

The strengths of this study included the application of quantitative and qualitative methods to evaluate devices using a usability evaluation framework that integrated the International Organization for Standardization Guidelines on Usability [18] and the Technology Acceptance Model [25,26], and the use of forest plots to examine within- and between-device variabilities to complement the use of other quantitative indices (ICC and κ). However, because of time constraints, we did not evaluate the variability of ECGs while patients were sitting, we did not explore in-depth views of patients, and most ECGs were acquired by 4 clinicians.

### Conclusions

The handheld 12-lead ECG device was comparable to routinely used conventional 12-lead ECG machine in its reliability and usability. The device’s small size, light weight, and wireless ECG transmission coupled with improved efficiency via training make the device a potential tool for clinician-led mass screening and remote assessment of patients. Patient body position should be included in the evaluation of device reliability because QTc lengthening secondary to standing offers diagnostic information. The user-centered evaluation framework utilized in this study could be applied to evaluate and better understand the acceptability and usability of new medical devices.

## Abbreviations

ECG: electrocardiography
ICC: intraclass correlation coefficient
QTc: corrected QT interval
